# A rare bilateral variant of the coracobrachialis muscle with supernumerary heads and coexisted variant branching patterns of the brachial plexus and the axillary artery

**DOI:** 10.1007/s00276-023-03088-w

**Published:** 2023-01-24

**Authors:** Dimitrios Filippou, Maria Piagkou, Konstantinos Natsis, Dimitrios Chytas, Georgia Kostare, George Triantafyllou, Evangelos Kostares, Christos Koutserimpas, Trifon Totlis, Marios Salmas, Vasilios Karampelias, George Tsakotos

**Affiliations:** 1 Department of Anatomy, School of Medicine, Faculty of Health Sciences, National and Kapodistrian University of Athens, 75 Mikras Asias Str., Goudi, 11527 Athens, Greece; 2 Department of Anatomy and Surgical Anatomy, School of Medicine, Faculty of Health Sciences, Aristotle University of Thessaloniki, Thessaloniki, Greece; 3 Basic Sciences Laboratory, Department of Physiotherapy, University of Peloponnese, Sparta, Greece; 4 Department of Orthopaedics and Traumatology, 251 Hellenic Air Force General Hospital of Athens, Athens, Greece

**Keywords:** Accessory head, Coracobrachialis muscle, Anatomic variation, Musculocutaneous nerve, Axillary artery, Nerve compression

## Abstract

**Purpose:**

The study report describes a rare bilateral variant of a six- and five-headed coracobrachialis muscle (CB). The musculocutaneous nerve (MCN) (bilaterally) and the median nerve (MN) lateral root (unilaterally) pierced CB heads, separating superficial from deep heads.

**Methods:**

The variant bilateral CB was identified in a 78-year-old formalin-embalmed male cadaver, derived from a body donation program after a signed informed consent.

**Results:**

*At the right side:* The 6-headed CB was pierced by the MCN, while the MN lateral root pierced the one superficial and deep head. CB was supplied by the lateral cord and the MCN. *At the left side:* A 5-headed CB was identified with three superficial distinct origins that fused into a common superficial head coursing anterior to MCN. The variant CB bilaterally (with 11 heads in total) coexisted with a MN variant formation, an atypical course of the MN lateral root through CB (right side), a connection of the MN lateral root with the MCN (left side) and a variant axillary artery branching pattern (bilaterally).

**Conclusions:**

Course and direction of the accessory CB heads may occasionally entrap the MCN and/or adjacent structures (brachial artery and MN). The MCN compression results in problems in the glenohumeral joint flexion and adduction, and tingling or numbness of the elbow joint, the forearm lateral parts and the hand.

## Introduction

Coracobrachialis muscle (CB), the smallest flexor of the anterior arm compartment, originates from the coracoid process and inserts medially, into the middle third of the humeral shaft. It is innervated by the musculocutaneous nerve (MCN), piercing the muscle heads, distinguishing the superficial from the deep heads [19], and is supplied by the brachial artery (BA) [20]. Koizumi [8, 9] demonstrated that the CB is composed of a superficial belly supplied by the lateral cord of the brachial plexus and a deep belly supplied by the MCN. The CB commonest morphological variant is the presence of one accessory head, while multiple accessory heads are quite unusual [19]. Occasional CB descriptions are referred to four [18] or even six [21] heads and different distal attachments [19]. The CB may also have accessory bundles inserting into the medial intermuscular septum, the medial epicondyle, the flexor carpi radialis, the brachialis, the brachioradialis or the pronator teres muscles [15]. Other variants include the CB longus, the CB brevis [16] and the coraco-capsularis muscle [20]. Course and direction of these accessory structures may occasionally entrap the median nerve (MN) and/or the BA [5]. The MCN may be compressed between CB heads [17, 19] leading to weakness of the anterior arm muscles and problems in the glenohumeral joint flexion and adduction. Tingling or numbness may occur, as the MCN provides sensory innervation to the elbow joint, the forearm lateral part, and the hand [18]. The current report describes a rare bilateral variant of a six- and five-headed CB originated from the coracoid process and the tendon of the biceps brachii (BB) short head. All heads are inserted into the medial surface of the humerus middle third. The MCN (bilaterally) and the MN lateral root (unilaterally) were piercing the CB heads, separating superficial from deep heads. Coexisted variants were also described. The systematic knowledge on the potential clinical consequences of such a rare bilateral finding is also discussed.

## Case report

**Fig. 1:**
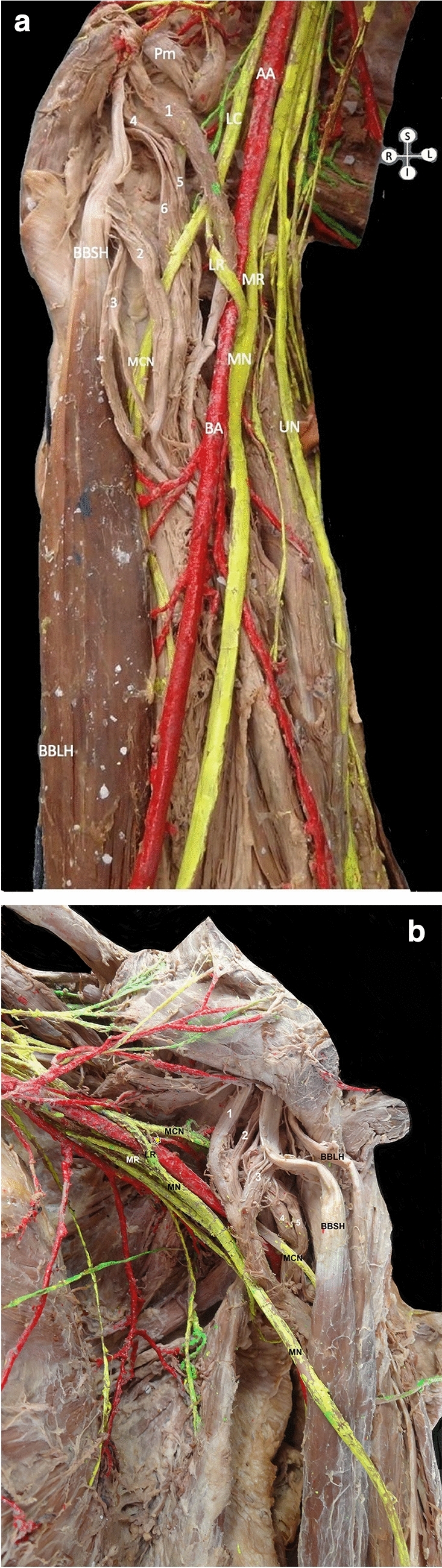
Dissection of the **A** six-headed (right side) and **B** five-headed (left side) coracobrachialis muscle (CB) variants. **A** Three superficial (1, 2 and 3) and 3 deep (4, 5 and 6) heads. Head 1 emanated from the coracoid process tip and heads 2, 3, 4, 5, and 6 originated from the tendon of the biceps brachii muscle short head (BBSH). Heads 4 and 5 are joined in a common deep head, which is fused with head 1 and the whole complex is inserted into the middle third of the humerus. The heads 3, 4, and 6 are inserted into the middle third of the humerus. The heads 1 and 2 are pierced by the musculocutaneous nerve (MCN), **B** three superficial (1, 2 and 3) and two deep heads (4 and 5). Heads 1 and 2 originated from the coracoid process and head (3) from the tendon of the BBSH. The three superficial heads fused into a common head, which was inserted into the middle third of humerus. The head (4) originated from the coracoid process base and the head (5) from the shoulder’s joint capsule. The two deep heads (4 and 5) formed a common deep head, that was inserted into the medial humeral shaft, superior to the insertion of the common superficial head. Both common heads are pierced by the MCN, MN-median nerve, BBLH-biceps brachii long head, LR-lateral root, MR-medial root, asterisk-connection branch between LR and MCN, UN-ulnar nerve, AA- axillary artery, BA-brachial artery, LC-lateral cord, and Pm-pectoralis minor, S-superior view, I-inferior view, L-left and R-right view

During the axillae and arms dissection of a formalin-fixed 78-year-old Greek male donated cadaver, a quite rare CB bilateral variant was detected. The cadaver was donated to the Anatomy Department of the Medical School of the National and Kapodistrian University of Athens, through the “Anatomical Gift Program” after written informed consent. The atypical CB had multiple accessory heads, bilaterally. The right-sided six-headed CB had three superficial (1, 2, and 3) and three deep heads (4, 5, and 6). All heads were pierced by the MCN, and the MN lateral root pierced heads 1, 4, 5, and 6. It seems that the CB was triplicated. The head 1 arose from the coracoid process tip, the heads 2 and 3, as well as the heads 4, 5, and 6 originated from the tendon of the BB short head. The heads 4 and 5 joined and before their insertion were fused with the head 1. The head 2 inserted into the middle third of the humeral shaft, after receiving a bundle from the head 3. The whole complex is inserted into the middle third of the humeral shaft. The six-headed CB was supplied by four branches of the lateral cord and from the MCN. The lateral cord penetrated CB and gave off the MCN and the MN lateral root, more distally than usual. The MCN passed between CB superficial and deep heads (Fig. 1A). At the contralateral side, a five-headed CB was identified. The CB had three superficial distinct origins that fused into a common superficial head coursing anterior to the MCN. The superficial heads 1 and 2 emanated from the coracoid process and joined. The superficial head 3 that originated from the tendon of the BB short head joined the superficial heads (1 and 2) complex and was commonly inserted medially, into the middle humeral third. The deep head 4 originated from the coracoid process base and joined the deep head, originating from the shoulder joint capsule. The deep heads 4 and 5 in common inserted into the medial humeral shaft, just above the common insertion of superficial heads (1, 2 and 3) (Fig. 1B). The variant CB coexisted with an MN variant formation, an atypical course of the MN lateral root through CB (right side), a connection of the MN lateral root with the MCN (left side) and a variant axillary artery (AA) branching pattern (bilaterally).

### Brachial plexus branches’ atypical formation, course, and other variants

**Fig. 2:**
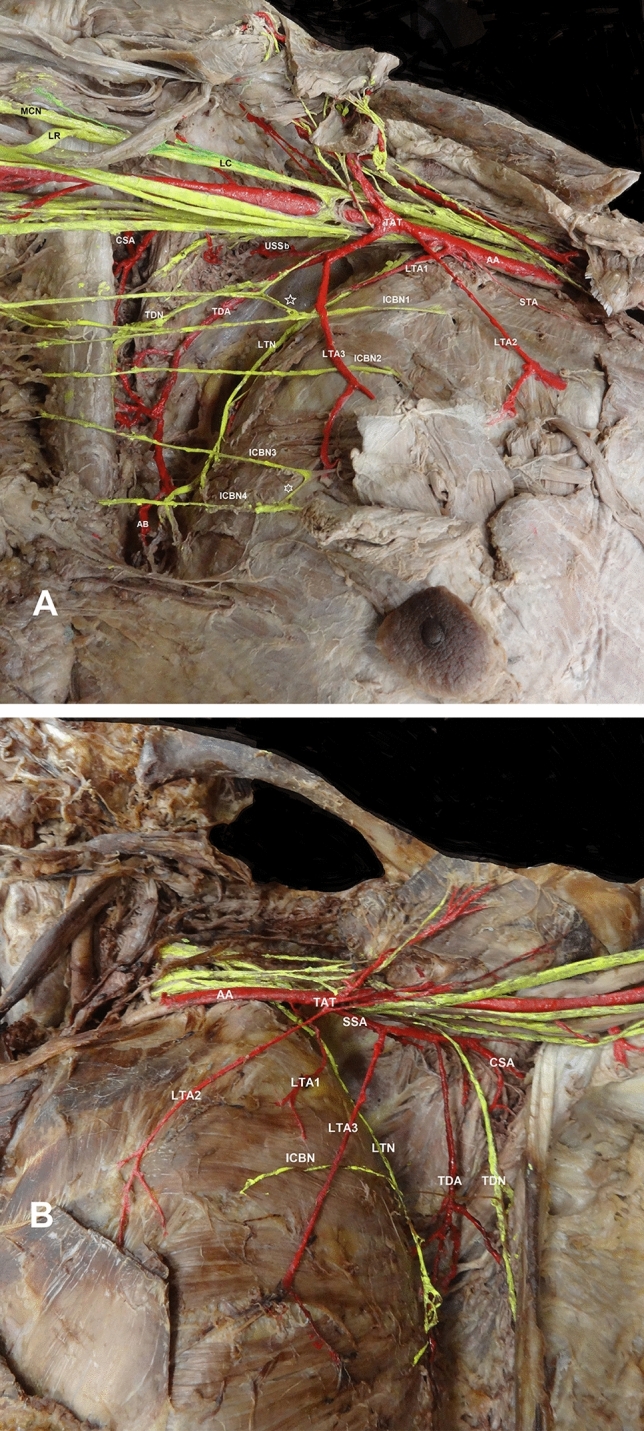
The bilateral variant of the axillary artery (AA) branching pattern **A** *Right side*, STA- superior thoracic artery, 1st lateral thoracic artery (LTA1) accompanied by the long thoracic nerve (LTN), 2nd and 3rd LTAs (LTA2 and LTA3), TAT-thoracoacromial arterial trunk in common with the LTA3, TDA-thoracodorsal artery accompanied by the thoracodorsal nerve (TDN), 1st, 2nd, 3rd and 4th intercostobrachial nerves (ICBN 1, 2, 3, and 4) and the interconnecting branch (*) of the ICBN1 with the medial cutaneous nerves of the arm and forearm, white big asterisk- connecting branch, CSA-circumflex scapular artery, USSb-upper subscapular branch, AB-angular branch, LC-lateral cord, LR-lateral root, and MCN-musculocutaneous nerve. **B**. *Left side*, three LTAs (LTA1, LTA2 and LTA3). The LTA1 partially was accompanied by the LTN, SSA-subscapular artery, TDA accompanied by the TDN

At the right side, the MN was formed distally, at the lower border of the latissimus dorsi muscle tendon, after the asymmetrical union of the lateral and medial roots (Fig. 2A). The medial cord was subdivided at a proximal level in relation to the lateral cord division. The four intercostobrachial nerves (branches of the 2^nd^, 3^rd^, 4^th^, and 5^th^ intercostal nerves) coursed anterior to the long thoracic nerve. On the left side, a connecting branch of the MCN to the MN lateral root was identified (Fig. 2B).

### The axillary artery atypical branching pattern

*At the right side*, three LTAs (LTA1, LTA2 and LTA3) and an abnormal trunk were identified. The LTA1 emanated from the AA 1^st^ part. The abnormal trunk, posterior to pectoralis minor muscle, gave off the LTA2, the thoracoacromial artery (TAA) and the subscapular artery that, 23.82 mm distally, divided into the LTA3, a branch for the subscapularis’ upper part and the thoracodorsal artery. The circumflex scapular artery and a trunk for the teres major and minor supply emanated from the AA 3^rd^ part, 4.1 cm distally (Fig. 2A).

*On the left side*, a symmetrical LTA triplication and an abnormal trunk were identified. Superior thoracic artery was absent, and at the 3^rd^ rib inferior border, the AA gave off the LTA1 and 0.5 cm below, the common trunk (TAA-2^nd^ LTA and 3 pectoral branches for the pectoralis major and minor supply). The subscapular artery arose medial to the TAA and gave off an upper branch for the subscapularis superior portion supply, the LTA3, the circumflex scapular artery, a branch for the teres major and the thoracodorsal artery (Fig. 2B). Overall cadaver status was without obesity or muscle atrophy. No signs of pathological conditions, trauma, or earlier surgery in the cadaver’s upper limbs were identified.

## Discussion

The cadaveric report describes a bilateral quite rare variant of a six- and five-headed CB with attachment on the medial surface of the humeral shaft. Szewczyk et al. [19] recorded two accessory CB heads in 7.9% and Olewnik et al. [18] found three accessory heads innervated by the MCN. Occasional CB descriptions with four [18] or even six [21] heads and different distal attachments [19] exist.

In the current report, the occurrence of such a bilateral complex structure is associated with developmental alterations. The aberrant muscle formations may be remnants of muscle primordia from different layers that failed to disappear or fuse to form a single muscle [3, 10]. Some of the brachial plexus fibers may follow an aberrant course, due to failure in differentiation. In the first stage, the flexor arm muscles are tightly connected in a pre-muscle mass. The three muscles become distinct structures when the embryo reaches a length of 14–16 mm. Probably, the CB additional heads are formed during this stage or later (at an embryonal length 11–19 mm) when the single mass divides into smaller parts recognized as CB supernumerary heads [1].

Due to CB high morphological variability, there is a possibility to identify new variants not previously described. CB accessory heads have also been associated with MCN variants. The MCN atypical course shows a wide variability [4, 14]. The MCN may not pierce CB (16.67%) [7]. The CB accessory heads may be combined with multiple neurovascular variants, as in the current case where triple LTAs were bilaterally and four intercostobrachial nerves were unilaterally detected. The rare occurrence of multiple LTAs (3.09%) is clinically significant [13]. Particularly the LTA and thoracodorsal artery points of origin [12] are important, since the arteries should remain intact during neck and breast surgery [13]. Concerning the intercostobrachial nerve, it should be preserved during the radical mastectomy to prevent sensory loss or dysesthesia in the upper arm area. Thus, its identification and possible variations, such as duplication, triplication and multiplication with their possible interconnections are quite important [11].

The presence of additional CB heads may have clinical implications, since the supernumerary head (s) may compress the MCN, especially when this nerve courses between two bellies [17, 19]. The MCN entrapment may complicate the glenohumeral joint flexion and adduction and cause tingling or numbness, as the MCN provides sensory innervation to the elbow joint, the forearm lateral parts and hand [17]. Entrapment at this level is extremely rare, but it may arise as a major issue, since both the anterior arm compartment and the forearm muscles may be affected leading to disabilities. Detailed imaging of the area with magnetic resonance imaging should be performed prior surgery, to evaluate the area variants, since such cases are extremely rare. Furthermore, shoulder surgery including osteotomy and coracoid process transfer in Bristow- Latarjet procedure, subcoracoid loops and muscle transfers requires muscles’ mobilization that are attached to the coracoid process [2]. Therefore, data concerning the level of MCN penetration into the CB will shed light on such surgical procedures, thus avoiding MCN iatrogenic lesions [4]. Intraoperative blunt trauma or elongation of MCN may lead to neurapraxia, therefore detailed planning of the procedure and the variants through preoperative imaging are of utmost importance for avoiding such complications.

CB has a definite clinical significance. During brachial plexus blockade, the CB serves as guide for AA identification [7]. It is well vascularized and represents a good choice as a transplant for treating long-standing facial palsy [3], as a graft for post-mastectomy reconstruction, and in both axillary and infraclavicular deformities [3]. In some cases, an accessory CB can lead to subcoracoid impingement and MCN, MN, or ulnar nerve palsy due to hypertrophy, injury, or brachial artery compression. Variant CB could also cause confusion during surgery and imaging evaluation [6]. The MCN atypical course would be of considerable value during axillary blocks and flap dissections, as well as in identifying and treating post-traumatic peripheral neuropathies and reconstructive nerve grafting following brachial plexus injury, coracoid process grafting and shoulder arthroplasty. Knowledge of the morphological variants of the shoulder area, prior to surgery, is crucial since many variants have also been reported at the acromion and the scapula. Thorough preoperative planning may minimize intraoperative “surprises” and, hence, complications.

## Study limitations

As the medical history of the dead man was unknown, the clinical impact of the identified variants could not be estimated. However, we believe that physicians and surgeons should be alert for the possible presence of such variants, because their occurrence may have clinical significance, as it was previously highlighted.

## Conclusions

To the best of our knowledge, such a combination of muscular and neurovascular variants has not been reported. The quite rare CB variant with multiple heads (11 in the number, 6 om the right and 5 on the left side) was identified bilaterally. The superficial heads were separated from the deep heads of the muscle complex by the MCN course. The six-headed CB was supplied by the lateral cord and the MCN. The variant CB coexisted with a MN variant formation, an atypical course of the MN lateral root through CB (right side), a connection of the lateral root with the MCN (left side) and a variant AA branching pattern (bilaterally) (with focus on LTA triplication and abnormal arterial trunks'formation) and an intercostobrachial nerve quadrupling.

## Data Availability

Data and material related to the report will be available with the corresponding author for further reference.

## Abbreviations

CB: Coracobrachialis muscle
MCN: Musculocutaneous nerve
MN: Median nerve
BA: Brachial artery
BB: Biceps brachii muscle
LTA: Lateral thoracic artery
TAA: Thoracoacromial artery
